# Genome-wide association of milk fatty acids in Dutch dairy cattle

**DOI:** 10.1186/1471-2156-12-43

**Published:** 2011-05-11

**Authors:** Aniek C Bouwman, Henk Bovenhuis, Marleen HPW Visker, Johan AM van Arendonk

**Affiliations:** 1 Animal Breeding and Genomics Centre, Wageningen University, P.O. Box 338, 6700 AH Wageningen, the Netherlands

## Abstract

**Background:**

Identifying genomic regions, and preferably individual genes, responsible for genetic variation in milk fat composition of bovine milk will enhance the understanding of biological pathways involved in fatty acid synthesis and may point to opportunities for changing milk fat composition via selective breeding. An association study of 50,000 single nucleotide polymorphisms (SNPs) was performed for even-chain saturated fatty acids (C4:0-C18:0), even-chain monounsaturated fatty acids (C10:1-C18:1), and the polyunsaturated C18:2*cis9,trans11 *(CLA) to identify genomic regions associated with individual fatty acids in bovine milk.

**Results:**

The two-step single SNP association analysis found a total of 54 regions on 29 chromosomes that were significantly associated with one or more fatty acids. *Bos taurus *autosomes (BTA) 14, 19, and 26 showed highly significant associations with seven to ten traits, explaining a relatively large percentage of the total additive genetic variation. Many additional regions were significantly associated with the fatty acids. Some of the regions harbor genes that are known to be involved in fat synthesis or were previously identified as underlying quantitative trait loci for fat yield or content, such as *ABCG2 *and *PPARGC1A *on BTA 6; *ACSS2 *on BTA 13; *DGAT1 *on BTA 14; *ACLY*, *SREBF1*, *STAT5A*, *GH*, and *FASN *on BTA 19; *SCD1 *on BTA26; and *AGPAT6 *on BTA 27.

**Conclusions:**

Medium chain and unsaturated fatty acids are strongly influenced by polymorphisms in *DGAT1 *and *SCD1*. Other regions also showed significant associations with the fatty acids studied. These additional regions explain a relatively small percentage of the total additive genetic variance, but they are relevant to the total genetic merit of an individual and in unraveling the genetic background of milk fat composition. Regions identified in this study can be fine mapped to find causal mutations. The results also create opportunities for changing milk fat composition through breeding by selecting individuals based on their genetic merit for milk fat composition.

## Background

The biosynthesis of bovine milk fat is a complicated process regulated by many genes belonging to several pathways [1]. Genetic analyses of bovine milk fatty acids have shown heritable variation. Short and medium chain fatty acids (C4:0 up to and including C16:0), which are synthesized de novo in the mammary gland, have moderate to high heritability [2,3]. Long chain fatty acids (i.e. C16:0 and higher) are derived from blood lipids that originate mainly from the diet and endogenous lipids, nevertheless, they have low to moderate heritability [2-4]. Identification of genomic regions, and preferably individual genes, responsible for genetic variation in milk fat composition will enhance the understanding of biological pathways involved in fatty acid synthesis and may point towards opportunities for changing milk fat composition via selective breeding. Candidate gene studies have shown that polymorphisms in *diacylglycerol O-acyltransferase 1 *(*DGAT1 K232A*) [5] and *stearoyl-CoA desaturase 1 *(*SCD1 A293V*) [6] have important effects on milk fat composition [7-12]. Many genes are involved in the biosynthesis of milk fat, and analyzing these candidate genes one by one in a candidate gene approach is not an option; therefore, quantitative trait loci (QTL) studies try to identify regions associated with milk fat composition to identify candidate genes that are worth considering.

In order to identify genomic regions involved in the biosynthesis of milk fat, Schennink et al. [13] and Stoop et al. [14] performed genome-wide linkage analyses of milk fatty acids and detected genome-wise significant QTL and several suggestive QTL. Other linkage studies have been performed for single chromosomes [15] or the fat composition of adipose tissue in beef cattle [15-18].

Recent developments in molecular genetics have made it possible to perform genome-wide association studies using thousands of single nucleotide polymorphism (SNP) markers to detect QTL. A genome-wide association study has higher power to detect QTL and provides more precise estimates of QTL locations compared to a linkage study. Some genome-wide associations for routinely evaluated traits in dairy cattle, such as milk production and fertility, have been published [19-22]. To the best of our knowledge, no genome-wide association study of milk fatty acids has been reported.

The aim of this study was to perform a genome-wide association analysis using 50,000 SNP markers to identify QTL for individual fatty acids in bovine milk. Associations were studied for even-chain saturated fatty acids (C4:0-C18:0), even-chain monounsaturated fatty acids (C10:1-C18:1), and the polyunsaturated fatty acid C18:2*cis9,trans11 *(CLA).

## Methods

### Phenotypes

The fat composition of winter milk samples from 1,905 first-lactation Dutch Holstein Friesian cows was available for this study. The cows were housed on 398 commercial farms throughout the Netherlands. At least three cows were sampled per farm. The cows were between 63 and 282 days in milk. The period of negative energy balance in early lactation was avoided by choosing cows over 63 days in lactation. The population consisted of five large paternal half-sib families from proven sires (200, 199, 195, 176, 101 daughters per sire) and 50 small paternal half-sib families from test-sires (10-24 daughters per sire), as well as 190 cows descending from 45 other proven sires (1-30 daughters per sire). The pedigree of the cows was supplied by CRV (Cooperative cattle improvement organization, Arnhem, the Netherlands) and consisted of 26,300 animals.

Milk fat composition was measured by gas chromatography. Many fatty acids were measured, but only the major fatty acids are reported here: even-chain saturated fatty acids C4:0 to C18:0, even-chain (*cis9*) monounsaturated fatty acids C10:1 to C18:1, and the polyunsaturated fatty acid CLA. The fatty acids were expressed in terms of weight-proportion of total fat weight (w/w%). In total, these fatty acids made up 89% of the total fat. Table 1 presents the mean, phenotypic standard deviation, and intra-herd heritability for the fatty acids included in this study. More detailed information about the population and phenotypes can be found in Stoop et al. [3].

**Table 1:** Descriptive statistics of milk fatty acids

Trait	Mean	σ_P_^1^	
C4:0	3.50	0.24	0.44
C6:0	2.22	0.14	0.47
C8:0	1.37	0.12	0.61
C10:0	3.03	0.35	0.72
C12:0	4.11	0.46	0.64
C14:0	11.61	0.78	0.62
C16:0	32.59	2.15	0.43
C18:0	8.72	1.18	0.24
C10:1	0.37	0.06	0.34
C12:1	0.12	0.02	0.38
C14:1	1.36	0.23	0.34
C16:1	1.44	0.30	0.44
C18:1	18.18	1.57	0.26
CLA	0.39	0.07	0.42

^1 ^Phenotypic standard deviation after adjusting for systematic environmental effects: days in milk, age at first calving, season of calving, and herd

Mean (w/w%), phenotypic standard deviation^1 ^(σ_P_), and intra-herd heritability () for the fatty acids of winter milk samples

### Genotypes

Blood samples were collected from the cows for DNA isolation. The cows were genotyped using a custom Infinium Array (Illumina, San Diego, CA, USA) designed by CRV. In total, 1,810 cows were successfully (call rate > 90%) genotyped. The cows were genotyped for 50,855 technically successful SNPs. The assumed map positions of the SNPs were based on the bovine genome assembly BTAU 4.0 [23]. From these 50,855 SNPs, a total of 776 SNPs could not be mapped to any of the *Bos taurus *(BTA) chromosomes and were assigned to BTA 0. In addition, 591 of the SNPs were located on the X chromosome. The SNPs on BTA 0 and the X chromosome were included in the study. The average distance between SNPs was 52,452 bp. Monomorphic SNPs (n = 245), SNPs with a genotyping rate < 80% (n = 383), and SNPs with a genotype frequency < 0.006 (1-9 observations for one of the genotype classes, SNPs with two genotype classes instead of three were included in the final marker set; n = 5,494) were discarded from the original SNP set of 50,855 SNPs, resulting in the final marker set of 44,733 SNPs used for the association analysis. Table 2 provides an overview of the number of SNPs available for the association study per chromosome.

**Table 2:** SNP information per *Bos Taurus *chromosome

BTA	SNP	Length (Mbp)	SNP interval (bp)	Monomorph	Genorate < 80%	Freq < 0.006
0^1^	776	-	-	1	6	84
1	3,011	160.91	53,371	4	25	355
2	2,451	140.64	57,333	20	19	269
3	2,342	127.13	54,212	12	11	260
4	2,300	124.09	53,930	11	20	239
5	2,215	125.78	56,788	5	18	214
6	2,844	122.39	43,050	12	25	334
7	2,017	111.67	55,392	8	14	209
8	2,131	116.93	54,818	15	15	243
9	1,860	108.05	58,090	14	20	206
10	1,911	106.10	55,406	14	11	202
11	2,193	110.01	50,187	18	12	239
12	1,512	85.22	56,324	6	14	147
13	1,689	84.00	49,732	11	12	155
14	2,122	81.29	38,272	9	17	222
15	1,446	84.23	58,130	5	7	169
16	1,455	77.83	53,454	8	12	183
17	1,561	76.40	48,942	5	14	159
18	1,282	66.04	51,429	3	8	131
19	1,452	65.13	44,826	5	7	147
20	1,479	75.41	50,985	3	9	156
21	1,246	69.08	55,440	5	15	130
22	1,256	61.75	49,161	4	10	139
23	1,169	53.27	45,570	7	10	107
24	1,296	64.93	50,141	10	13	159
25	1,256	43.44	34,617	6	9	109
26	1,131	51.00	45,097	7	6	152
27	933	48.73	52,280	3	13	117
28	899	46.01	51,184	6	5	94
29	1,029	51.78	50,371	6	4	109
X	591	88.46	149,940	2	2	55

Total	50,855	2,628		245	383	5,494

^1 ^unmapped SNPs

Total number of SNPs, map length, average SNP interval, number of monomorphic SNPs, number of SNPs with a genotyping rate (genorate) < 80% and number of SNPs with a genotype frequency (freq) < 0.006 for all *Bos Taurus *(BTA) chromosomes.

### Statistical analysis

For the association study, both phenotype and genotype information was available for 1,706 individuals. A two-step single SNP association analysis was performed. In the first step, the genome was screened for interesting regions using a general linear model. In the second step, the interesting regions were verified using an animal model.

In the first step, a genome-wide association study was performed with a general linear model using the R package 'SNPassoc' [24]. In this step, the analyzed phenotypes were pre-adjusted for systematic environmental effects, and the general linear model accounted for the SNP effect and the effect of sire. The general linear model used in the first step was:(1)

where y* was the phenotype adjusted for the systematic environmental effects; sire was the fixed effect of sire; SNP was the fixed effect of SNP genotype; and e was the random residual. Sire effect was included in the SNPassoc model to account for paternal half-sib relations. Phenotypes were adjusted for days in milk, age at first calving, calving season, and herd. Adjusted phenotypes were obtained from the phenotypes of 1,905 cows using an animal model in ASReml [25]:(2)

where y was the (unadjusted) phenotype; μ was the overall mean; dim was the covariate describing the effect of days in milk; afc was the covariate describing the effect of age at first calving; season was the fixed effect of the class of calving season (June-Aug 2004, Sept-Nov 2004, or Dec 2004-Jan 2005); scode was the fixed effect accounting for differences in genetic level between groups of proven bull daughters, young bull daughters, and other bull daughters; herd was the random effect of herd, distributed as N(0, I ), with identity matrix I and herd variance ; animal was the random additive genetic effect of the individual, distributed as N(0, A ), with the additive genetic relationship matrix A and the additive genetic variance ; and e was the random residual, distributed as N(0, I ), with identity matrix I and residual variance .

The genome-wide false discovery rate (FDR) was controlled according to the method described by Storey and Tibshirani [26], by separately calculating the genome-wide FDR based on the *P*-values from the general linear model for each trait using the R package 'qvalue'. Associations with a genome-wide FDR < 0.05 for the general linear model were considered significant.

The first step was performed to identify interesting regions, which were then further analyzed with an animal model to account for all relationships among individuals. Including a polygenic effect and accounting for genetic relationships would be more appropriate [27]. The model including a polygenic effect is computationally demanding when analyzing many traits, SNPs, and animals; therefore, in the second step we only analyzed the regions that contained multiple SNPs that were significant in the first step.

A region started at the first significant SNP on a chromosome that was followed by an additional significant SNP within 10 Mbp; the region was extended as long as another significant SNP occurred within 10 Mbp from the previous one and ended at the last significant SNP that was not followed by another significant SNP within the next 10 Mbp. Thus, a region contained at least two significant SNPs. More than one region could be present on the same chromosome when there were groups of significant SNPs located within 10 Mbp from each other but further than 10 Mbp from the other region(s) on the chromosome. The 10 Mbp distance between significant SNPs is rather large, but it was chosen to prevent having many small regions on one chromosome, each containing a small number of significant SNPs.

In the second step, all SNPs in regions with significant effects were analyzed using animal model (2) extended with an SNP effect in ASReml [25]. In this model the phenotypes were simultaneously adjusted for systematic environmental effects, for all genetic relationships among individuals, and for the SNP genotype. Associations with a -log_10_(*P*-value) ≥ 3 were considered significant.

The genetic variance explained by an SNP was calculated from the estimated genotype effects from animal model (2) extended with an SNP effect and the observed genotype frequencies. The result was expressed as a percentage of the total additive genetic variance. These percentages can be overestimated, especially when the effect of an SNP is small, this is due to the so called Beavis effect [28]. The percentage of the total additive genetic variance explained by the most significant SNP per trait per region is reported. The most significant SNP can differ per trait for a region associated with multiple traits.

## Results

**Figure 1:**
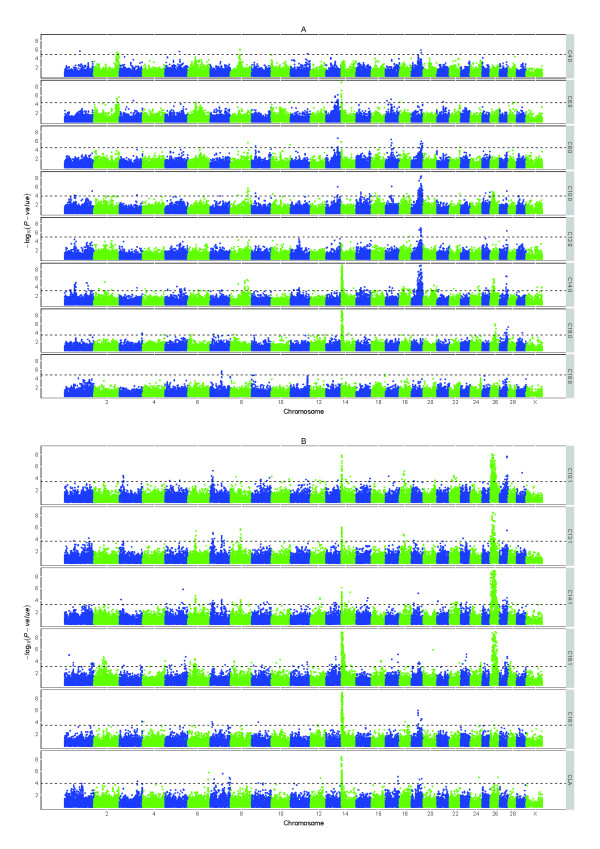
**Genome-wide association plots for milk fatty acids**. Genome-wide plots of -log_10 _(*P*-values) (y-axis) for association of loci with saturated fatty acids (A) and unsaturated fatty acids (B) analyzed with a general linear model. The genomic position is represented along the x-axis and chromosome numbers are given on the x-axis. The dashed horizontal lines represent the 0.05 false discovery rate thresholds. The y-axis are cut off at -log_10_(*P*-value) of 9.

In the first step of the single SNP association study, all SNPs were analyzed using a general linear model. The analysis resulted in many significant (FDR < 0.05) associations between SNPs and the studied fatty acids. Figure 1 shows the genome-wide plots of -log_10_(*P*-values) for all of the studied fatty acids. All analyzed fatty acids showed significant associations with at least one genomic region.

From the results of the general linear model, a total of 64 significant regions were defined, affecting one or more (up to 10) traits. Significant associations with SNPs were detected on all chromosomes; however, on BTA 29 we identified only a single SNP associated with a trait instead of multiple SNPs in a region. Therefore, no regions were defined on BTA 29. These 64 regions were analyzed with an animal model and 54 remained significant at -log_10_(*P*-value) ≥ 3. Thus, most of the regions identified with the general linear model were confirmed with the animal model. The correlation between *P*-values in both analyses was 0.90. All results mentioned hereafter originate from the animal model and concern the 54 regions that remained significant at -log_10_(*P*-value) ≥ 3 with the animal model.

Significant associations were detected with several regions for most of the fatty acids, especially C14:0 (19 regions) and C16:1 (18 regions), whereas C18:0 and C12:0 were associated with only one region (Table 3). The X chromosome showed significant association with C14:0. Table 3 shows that several regions affect more than one trait. In particular, the medium chain unsaturated fatty acids were often associated with the same region.

**Table 3:** Regions significantly (-log_10_(*P*-value ≥ 3)) associated with fatty acids and the percentage of the total additive genetic variance explained

Region	Start (Mbp)	End (Mbp)	# SNP in region	Trait														#Traits/region
					
				C4:0	C6:0	C8:0	C10:0	C12:0	C14:0	C16:0	C18:0	C10:1	C12:1	C14:1	C16:1	C18:1	CLA	
1a	24.8	24.8	2												3.3			1
1b	54.1	62.8	148				0.9		2.0									2
1c	121.5	129.9	120						3.0									1
1d	146.4	161.0	304												2.1			1
2a	27.6	27.7	3						1.8									1
2b	44.3	69.5	351												2.7			1
2c	126.1	139.5	257	4.0	3.3													2
3a	22.2	25.1	55									4.1						1
3b	125.2	126.9	39							2.9						5.3		2
4	121.8	123.7	30												2.2			1
5a	65.9	65.9	3						1.5									1
5b	81.9	99.9	275						2.7					4.8				2
5c	107.5	113.6	104						1.8						2.7			2
6a	31.4	45.2	630										4.3	1.3	3.1			3
6b	111.2	115.6	81														4.2	1
7a	10.7	23.8	206									4.1	3.9	3.3		4.3		4
7b	62.4	64.2	46						2.0		7.4		3.4	3.0				4
7c	100.3	110.7	166														3.1	1
8a	20.5	21.9	28												2.7			1
8b	52.7	58.4	104	4.5									4.2					2
8c	76.9	101.5	406				1.3		2.4									2
9a	21.4	21.6	2				1.1											1
9b	36.7	36.7	3													3.9		1
9c	48.8	50.4	25									3.9						1
9d	66.6	67.0	4											4.8				1
10a	8.3	8.3	3				1.2											1
10b	98.8	100.2	34									2.8						1
11	35.2	48.0	218						2.3									1
12	52.4	60.1	116									2.2		2.8				2
13	49.5	71.5	377		3.0	2.2	1.9							4.4	3.3			5
14a	0.0	26.3	1121		4.9	2.2			17.7	39.6		6.2	4.8	4.5	34.8	61.9	12.2	10
14b	40.0	40.8	7							3.7								1
15a	20.5	27.0	121				1.2		1.8									2
15b	60.7	65.1	63											3.4				1
15c	72.3	77.7	103									3.8						1
16	53.2	53.5	9												2.3			1
17a	31.4	34.3	43			2.9	1.9											2
17b	68.7	68.7	3												3.5		3.0	2
18a	14.1	29.5	305						1.8			3.1	3.5					3
18b	53.6	54.9	15						1.9									1
19a	6.1	6.1	3												2.3			1
19b	32.7	61.8	529	6.5		3.0	4.4	5.3	13.8						3.0	7.1	3.9	8
20	72.9	73.6	18						1.6									1
21	5.8	5.8	4											3.0				1
22	8.9	42.4	593									3.9		3.5				2
23	26.3	31.7	141												2.9			1
24a	40.4	49.1	142						1.3									1
24b	56.4	58.6	27												2.2			1
25	42.9	42.9	2				1.5											1
26	2.5	41.2	724				3.3		4.5	4.8		35.4	20.5	67.8	23.2			7
27	28.7	48.3	358						2.7	3.7		7.6	4.1	4.4	3.2			6
28a	6.8	13.2	116												2.8			1
28b	19.7	19.7	2											2.9				1
X	85.2	86.4	16						2.0									1

Sum			8,605	14.9	11.2	10.3	18.9	5.3	68.7	54.7	7.4	77.1	48.7	114.0	102.3	82.6	26.5	
All SNPs simultaneous in model^2^				14.9	10.2	10.9	16.3	5.3	41.8	53.9	7.4	62.0	45.2	97.4	79.2	78.6	24.1	
# Regions/trait				3	3	4	10	1	19	5	1	11	8	14	18	5	5	107

^1 ^A region starts at the first significant SNP and proceeds if the next significant SNP is positioned within the next 10 Mbp on the same chromosome, ending at the position of the last significant SNP matching this requirement, with a minimum of 2 significant SNP per trait in a region. The number of the region stands for the BTA number plus an a, b, c or d indicating different regions within a BTA.

^2 ^Percentage of the total additive genetic variance explained by all the most significant SNPs per region together. This was analyzed with the animal model and all the most significant SNPs per region simultaneous in the model.

Percentage of total additive genetic variance explained by the most significant SNP associated with the trait per region, analyzed with an animal model.

Sixteen of the unmapped SNPs showed significant associations with one or more of the studied fatty acids. Comparing the sequence of these significant unmapped SNPs against genome assembly UMD 3.0 [29] identified 14 of them as mapping to regions 14a, 19b, and 26, which were already identified as being associated with the traits. The other two unmapped SNPs (*ULGR_BTA-38166 *and *ULGR_BTA-38169*) were significantly associated with C10:0 and located at 24.2 Mbp on BTA 16 according to the UMD 3.0 genome assembly [29].

For each region, we estimated the variance explained by the most significant SNP for each trait. Within a region, this most significant SNP can be a different SNP per trait. The percentage of total additive genetic variance explained by the most significant SNP per region ranged from 0.9 for C10:0 on BTA 1 to 61.9 for C18:1 on BTA 14 (Table 3). These percentages can be overestimated, especially when the effect of an SNP is small, this is due to the so called Beavis effect [28]. The QTL with large effects, such as region 14a and 26, are less likely overestimated.

Three chromosomes showed highly significant regions, which were associated with multiple fatty acids: region 14a showed significant associations with 10 traits, region 19b with 8 traits, and region 26 with 7 traits. The region 14a harbors *DGAT1 *and region 26 harbors *SCD1*; these genes are known to affect fat composition [7-12]. These regions explained a large portion of the total additive genetic variation. The results also showed that additional regions had an effect on single fatty acids or a certain group of fatty acids.

In this section the SNP showing the strongest association of the whole genome for each trait is reported. Strongest associations for C14:0 (-log_10_(*P*-value) = 36.6), C16:0 (-log_10_(*P*-value) = 51.3), and C16:1 (-log_10_(*P*-value) = 54.1) were found with two SNPs (*ULGR_SNP_AJ318490_1b *and *ULGR_SNP_AJ318490_1c*), located at 0.4 Mbp on BTA 14, that are responsible for the *DGAT1 K232A *polymorphism. For C6:0 (-log_10_(*P*-value) = 8.4), C18:1 (-log_10_(*P*-value) = 48.6), and CLA (-log_10_(*P*-value) = 15.8) the *DGAT1 K232A *SNPs were the most significant SNPs immediately after an unmapped SNP (*ULGN_SNP_AJ318490_2*). After comparing this unmapped SNP sequence against the sequence of *DGAT1*, we identified it as an SNP located in *DGAT1 *that is in high LD (r^2 ^= 0.99) with the *DGAT1 K232A *SNPs. The strongest associations for C10:1 (-log_10_(*P*-value) = 41.1), C12:1 (-log_10_(*P*-value) = 22.8), and C14:1 (-log_10_(*P*-value) = 80.0) were found with an unmapped SNP (*ULGR_SNP_SCD*). Also, C16:1 showed strong association (-log_10_(*P*-value) = 34.8) with this unmapped SNP on BTA 26, but C16:1 showed the strongest association with the *DGAT1 K232A *SNPs on BTA 14 as mentioned above. After comparing this unmapped SNP sequence against the sequence of *SCD1*, we identified it as the SNP underlying the *SCD1 A293V *polymorphism. Although *SCD1 *was not mapped on genome assembly BTAU 4.0, it was mapped on genome assembly UMD 3.0 at 21.1 Mbp on BTA 26 [29]. The strongest associations for C10:0 (-log_10_(*P*-value) = 11.0) and C12:0 (-log_10_(*P*-value) = 9.7) were found with an SNP (*ULGR_MARC_10099_486*) located at 52.5 Mbp on BTA 19. The strongest association for C4:0 (-log_10_(*P*-value) = 6.6) was found with an SNP (*ULGR_BTA-45866*) located at 54.6 Mbp on BTA 19, and the strongest association for C8:0 (-log_10_(*P*-value) = 6.2) was found with an SNP (*ULGR_BTA-45847*) located at 55.1 Mbp on BTA 19. Also, C14:0 showed strong association (-log_10_(*P*-value) = 22.0) with an SNP (*ULGR_BTA-45758*) on BTA 19 located at 52.1 Mbp, but C14:0 showed the strongest association with the *DGAT1 K232A *SNPs on BTA 14 as mentioned above. The strongest association for C18:0 (-log_10_(*P*-value) = 5.8) was found with an SNP (*ULGR_BTA-28678*) located at 64.2 Mbp on BTA 7.

## Discussion

This study is the first to report a genome-wide association study of bovine milk fatty acids. A two-step single SNP association analysis was performed. In the first step, the genome was screened for interesting regions using a general linear model. In the second step, the interesting regions were verified using an animal model. The animal model accounted for all relationships between individuals in the pedigree, whereas the general linear model accounted only for the paternal half-sib structure of the phenotyped cows. Ignoring relationships between individuals can cause false positive associations [30]; therefore, the animal model was applied to verify the results from the general linear model.

The results showed that *DGAT1 *and *SCD1 *are highly associated with several of the fatty acids. The results also showed that, for some traits, other regions were more significantly associated, such as BTA 19 for some short and medium chain saturated fatty acids. In addition, many other regions were associated with fatty acids, but with smaller effects.

Schennink et al. [13] and Stoop et al. [14] reported a linkage study of milk fat composition using some of the same data used in the present study. In the linkage study, 1,341 SNPs were genotyped in 849 cows and their seven sires. Schennink et al. [13] and Stoop et al. [14] detected genome-wide significant QTL on BTA 6, 14, 19, and 26 for fatty acids included in the present study. The QTL on BTA 14, 19, and 26 were confirmed in our study. Our results suggest a QTL on BTA 6 for C6:0 and C8:0 (Figure 1A), but for C6:0 only one SNP exceeded the FDR threshold, and for C8:0 none of the SNPs exceeded the FDR threshold. Therefore, this region was not included in the animal model analysis in our second step. Given the quite stringent threshold, this region is still likely to harbor a QTL for C6:0 and C8:0.

The suggestive QTL found by Schennink et al. [13] and Stoop et al. [14] on BTA 2, 13, 14, 17, 19, and 26 was also confirmed in our study. Other suggestive QTL reported in the studies could not be confirmed, though in some cases a QTL was indicated, but this did not pass the threshold. In addition, several novel regions significantly associated with fatty acids were detected in our study but not reported by Schennink et al. [13] and Stoop et al. [14].

Some studies of the fat composition of adipose tissue in beef cattle were confirmed by our findings regarding milk fat composition. This result indicates that these regions are not unique for milk fat composition. Our findings confirmed the QTL detected by Morris et al. [18] in subcutaneous fat from beef cattle: C16:1 on BTA 1; C14:0, C16:1, and C18:1 on BTA 19; C14:0 and C14:1 on BTA 26; and C14:0 on BTA 27. Our findings confirmed the QTL detected by Abe et al. [17] in back fat, intermuscular fat, or intramuscular fat from beef cattle: C14:0 and C18:1 on BTA 19. Our findings confirmed the QTL detected by Alexander et al. [16] in the longissimus dorsi of beef cattle: CLA on BTA 7. Our findings confirmed the QTL detected by Uemoto et al. [31] in intramuscular adipose tissue from beef cattle: C18:1 on BTA 19.

### Major regions

Three major regions were detected in this genome-wide association study (regions 14a, 19b, and 26), with significant effects on milk fat composition. These regions showed highly significant associations with several fatty acids. The regions on BTA 14 and 26 are regions that have been studied previously, and our results confirm the earlier findings [5-12]. The region on BTA 19 has not been studied extensively in relation to milk fat composition, but it harbors a number of candidate genes involved in fatty acid synthesis.

The region on BTA 14 showing an association with C6:0, C8:0, C14:0, C16:0, C10:1, C12:1, C14:1, C16:1, C18:1, and CLA is the region harboring *DGAT1*, which is known to influence milk production traits [5] and milk fat composition [9,10,12]. For all of these traits, except C10:1, C12:1, and C14:1, the two most significant SNPs on BTA 14 (located at 0.4 Mbp) were the two SNPs corresponding to the dinucleotide substitution of *DGAT1 *resulting in a *K *to *A *amino acid substitution (*DGAT1 K232A*). The *K *allele is associated with larger fractions of C6:0, C8:0, C16:0, and C16:1, and with smaller fractions of C14:0, C18:1, and CLA.

For C10:1 and C14:1, the most significant SNP (*ULGR_BTC-068225*) was located at 3.0 Mbp, and for C12:1 the most significant SNP (*ULGR_BTC-067423*) was located at 3.7 Mbp. However, for C10:1 and C12:1, the *DGAT1 K232A *SNPs were also significant. After correcting the phenotypes for the effect of the *DGAT1 K232A *polymorphism, the -log_10_(*P*-values) of these most significant SNPs decreased from 6.50 to 2.30 for C10:1, from 5.44 to 2.52 for C12:1, and from 4.63 to 2.40 for C14:1. The LD between these most significant SNPs and the *DGAT1 K232A *SNPs was moderate (r^2 ^= 0.26 and 0.31). Although the associations of C10:1, C12:1, and C14:1 were just below the significance threshold after correcting for the *DGAT1 K232A *genotype, the findings still suggest that an additional QTL may be present for the medium chain unsaturated fatty acids on BTA 14.

The region on BTA 26 that showed an association with C10:0, C14:0, C16:0, C10:1, C12:1, C14:1, and C16:1 is the region harboring *SCD1*, which is known to be associated with the desaturation of fatty acids [7,8,10,11]. For all of these traits, except C16:0, the most significant SNP on BTA 26 corresponded to the nucleotide substitution in *SCD1 *that causes an *A *to *V *amino acid substitution (*SCD1 A293V*) at 21.1 Mbp. The *A *allele was associated with larger fractions of C10:1, C12:1, and C14:1, and with smaller fractions of C10:0, C14:0, and C16:1. Thus, the *A *allele resulted in more C10:1 and C14:1 at the cost of C10:0 and C14:0. A similar effect was found for C12:0 and C12:1, though C12:0 was not significantly associated with the SNP. The opposite effect was found for C16:0 and C16:1; the *A *allele resulted in less C16:1 and more C16:0, though C16:0 was not significantly associated with the SNP. The SCD1 gene codes for the SCD enzyme, which desaturates saturated fatty acids to Δ9 unsaturated fatty acids in the mammary gland [32]. The association of this SNP with, and its effects on, the medium chain unsaturated fatty acids and their equivalent saturated fatty acids is, therefore, in agreement with the function of the enzyme. The associations we identified for the medium chain unsaturated fatty acids confirm previous studies on the effect of the *SCD1 A293V *polymorphism on milk fatty acids [8,10,11]. The associations we identified for the medium chain saturated fatty acids confirm only the results of Schennink et al. [10], who used the same population as the present study.

The *SCD1 A293V *SNP was not significant for C16:0 (-log_10_(*P*-value) = 1.09), which also confirms previous studies [7,10,11]. The most significant SNP for C16:0 on BTA 26 was located at 28.8 Mbp. This SNP was not in LD with the *SCD1 A293V *SNP (r^2 ^= 0.08), and correcting for *SCD1 A293V *had little effect on the significance of the SNP associated with C16:0 (-log_10_(*P*-value) decreased from 5.52 to 4.46). Also, one allele of this SNP is associated with larger fractions of C10:1, C12:1, C14:1, and C16:0, and with smaller fractions of C10:0, C12:0, C14:0, and C16:1, suggesting that it has something to do with desaturation, but it was only significantly associated with C16:0. We did not identify obvious candidate genes in this region.

The region on BTA 19, at 32.7-61.8 Mbp, showed associations with C4:0, C8:0, C10:0, C12:0, C14:0, C16:1, C18:1, and CLA, i.e. mainly with the short and medium chain saturated fatty acids and with the long chain unsaturated fatty acids. No significant effects were found for C6:0, but a QTL was indicated below the threshold in the region on BTA 19 (Figure 1A). Morris et al. [15] performed a linkage analysis of milk fatty acids on BTA 19, detecting QTL for C8:0, C10:0, C12:0, C14:0, C18:1, and C18:2, which was confirmed by our findings and suggested *fatty acid synthase *(*FASN*, at 52.2 Mbp) as a candidate gene responsible for the observed effect. In addition to *FASN*, several other genes located within the region on BTA 19 are involved in the biosynthesis of milk fat, including *sterol regulatory element binding transcription factor 1 *(*SREBF1*, at 35.7 Mbp), *ATP citrate lyase *(*ACLY*, at 43.4 Mbp), *signal transducer and activator of transcription 5A *(*STAT5A*, at 43.7 Mbp), and *growth hormone *(*GH*, at 49.7 Mbp). These genes are all candidate genes because SNPs in the whole region showed an association with the traits, perhaps in LD with mutations in genes not captured by our marker set. The strongest association was found near *FASN*, but also near some other candidate genes as discussed below.

The region on BTA 19 was strongly associated with C14:0 and explained a large portion of the total additive genetic variation of C14:0. The SNP most significant for C14:0 (-log_10_(*P*-value) = 22.04) was also the most significant SNP for C18:1 (-log_10_(*P*-value) = 4.52) and located at 52.1 Mbp on BTA 19 (*ULGR_BTA-45758 *in *LOC518878*), which is 71,862 bp from *FASN*. This SNP also showed significant effects on C4:0 (-log_10_(*P*-value) = 3.57), C10:0 (-log_10_(*P*-value) = 7.01), C12:0 (-log_10_(*P*-value) = 5.96), and CLA (-log_10_(*P*-value) = 3.67), whereas the association with C8:0 (-log_10_(*P*-value) = 2.96) was just below the threshold. The effects of SNPs on C8:0, C10:0, C12:0, and C14:0 were in opposite direction of the effects on C4:0, C16:1, C18:1, and CLA. Fatty acid synthase (encoded by *FASN*) is a multi-enzyme system involved in de novo fatty acid synthesis. The SNP effects suggest that less C4:0 and more C8:0, C10:0, C12:0, and C14:0 are produced by fatty acid synthesis, or vice versa, which is in line with the function of FASN. Three SNPs in *FASN *were included in the marker set used in our study; however, two of them (*FASN*_*16009 *_and *FASN*_*763*_) were monomorphic for our population. The third SNP, *FASN*_*17924*_, showed association with C14:0 (-log_10_(*P*-value) = 3.05). Schennink et al. [33] also studied *FASN*_*16024*_, finding a significant association with C14:0 for the same population as in our study. Morris et al. [15] did not find significant associations between *FASN*_*17924 *_and C14:0, but did find a significant association between C14:0 and *FASN*_*15531 *_and *FASN*_*15603*_.

A haplotype of five *FASN *SNPs has been shown to be significantly associated with C6:0, C8:0, C10:0, C12:0, C14:0, and C18:1 in Friesian-sired cows [15], which are almost the same traits for which we found an association in the region, though not specifically with SNPs in *FASN*. This finding suggests a QTL in this region with an effect on short and medium chain fatty acids and long chain unsaturated fatty acids, but whether it is actually *FASN *that causes the association remains unclear. Our genome-wide scan showed that an SNP outside of *FASN *is the most significant SNP for C14:0 and was also associated with some of the other traits. This SNP showed very strong association with C14:0, whereas the association of C14:0 with the SNP in *FASN *was just barely significant. Candidate gene studies have shown that *FASN *is mainly associated with C14:0, but we found associations with additional traits, similar to the haplotype findings of Morris et al. [15]. Perhaps the causal mutation is located outside of *FASN *and is mainly the effect on C14:0 strong enough to be detected by SNPs in LD with this mutation.

This region on BTA 19 also harbors *GH*, and SNPs in this gene have been associated with milk production traits, including fat yield [34]. One SNP in our marker set was located in exon 5 of *GH *(*GH-D30713-299*) and showed significant association with C18:1 (-log_10_(*P*-value) = 3.81). The neighboring SNP showed even more associations: with C8:0 (-log_10_(*P*-value) = 3.48), C10:0 (-log_10_(*P*-value) = 5.13), C12:0 (-log_10_(*P*-value) = 3.86), and C14:0 (-log_10_(*P*-value) = 7.41).

No SNPs were located in the other candidate genes on BTA 19. The SNP showing the strongest association with C16:1 was located at 43.8 Mbp on BTA 19 (*BFGL-NGS-111365*), which is 13,873 bp from *STAT5A*. The SNPs neighboring *ACLY *and *STAT5A *showed significant associations with several of these traits, especially C14:0. All SNPs in the regions seem to be detecting the same effect, which is strongest with C14:0. Although previous studies suggested *FASN *as the candidate gene for association, which of the candidate genes causes the effect remains debatable. The causal mutation might even be in a gene not considered here.

### Additional regions

In addition to the three major regions mentioned above, many additional regions showed associations with fatty acids (see Figure 1). We will not discuss all of the regions in this paper, but what follows are some select regions that showed association with three or more traits.

On BTA 6, region 6a was associated with C12:1, C14:1, and C16:1 (Table 3). The SNP effects were in the same direction for all three fatty acids. This region harbors the genes *ATP binding cassette, subfamily G, member 2 *(*ABCG2*, 37.4 Mbp) and *peroxysome proliferator-activated receptor-gamma coactivator-1alpha *(*PPARGC1A*, 44.8 Mbp). The SNPs most significantly associated with C12:1 (-log_10_(*P*-value) = 5.03, at 44.3 Mbp), C14:1 (-log_10_(*P*-value) = 4.39, at 41.2 Mbp), and C16:1 (-log_10_(*P*-value) = 4.63, at 40.2 Mbp) were located between these candidate genes. *ABCG2 *has been associated with milk fat yield and percentage [35,36]. One SNP in our marker set was located in *ABCG2 *and showed no significant associations with the studied fatty acids. *PPARGC1A *has been associated with milk fat yield in German Holsteins [37], but this was not confirmed by Khatib et al. [38] in two larger American Holstein populations. Up to 10 SNPs in our marker set were located in *PPARGC1A*, but none of these were significantly associated with C12:1, C14:1, or C16:1. However, one of the 10 SNPs in *PPARGC1A *showed an association with C16:1, which was just below the significance threshold (-log_10_(*P*-value) = 2.86). In a candidate gene study, Schennink et al. [33] found a significant association in the same population as in the present study for two SNPs in *PPARGC1A *that were not included in our marker set: *PPARGC1A*_*1790+514 *_with C16:1 and *PPARGC1A*_*1892+19 *_with C14:1. Our genome-wide scan indicates that not *PPARGC1A*, but another region has the strongest effect on the unsaturated medium chain fatty acids.

On BTA 7, two regions showed an association with several fatty acids: region 7a associated with C10:1, C12:1, C14:1, and C18:1; and region 7b associated with C14:0, C12:1, C14:1, and C18:0 (Table 3).

Region 7a showed an association with almost all unsaturated fatty acids. In general, the SNP effects on C18:1 were in the opposite direction of the SNP effects on C10:1, C12:1, and C14:1. SNPs in this region were either associated with C10:1, C12:1, and C14:1, or with C18:1, suggesting one QTL for the medium chain unsaturated fatty acids and another for C18:1. The SNP effects on C10:0, C12:0, and C14:0 were in the same direction as the effects on C10:1, C12:1, and C14:1, but they were not significant. The SNP effects on C18:0 were in the same direction as the effects on C18:1, but they were not significant. Although only unsaturated fatty acids were significantly associated with this region, the SNP effects suggested that this QTL has nothing to do with desaturation because the SNP effects on unsaturated fatty acids were in the same direction as the effects on their saturated equivalents.

Region 7b showed associations with C14:0, C18:0, C12:1, and C14:1. The most significant SNP for each trait was different, but they were located in the same neighborhood, around 64.0-64.1 Mbp, and were in high LD with one another (r^2 ^= 0.53-0.97). This finding indicates the likelihood of one QTL in this region with an effect on these four traits. The effects of these most significant SNPs were in the opposite direction for C18:0 compared to C14:0, C12:1, and C14:1. The SNP effects on C10:0 and C12:0 were in the same direction as the effects on C14:0, C12:1, and C14:1, but they were not significant. The SNP effects on C16:0, C18:1, and CLA were in the same direction as the effects on C18:0, but they were not significant. These SNP effects suggest that this QTL has something to do with a trade-off between long chain fatty acids and de novo synthesis of medium chain fatty acids. No candidate genes were located in this region.

On BTA 13, region 13 was associated with C6:0, C8:0, C10:0, C14:1, and C16:1 (Table 3). This region confirms the QTL for C6:0, C14:1, and C16:1 detected by Stoop et al. [14], who also found that C8:0 and C10:0 showed a QTL around the same position as C6:0, but these QTL were just below the threshold and, therefore, not reported. For C6:0, C8:0, and C10:0, the same SNP, located at 64.8 Mbp (*ULGR_SNP_BES11_Contig346_1209*), was the most significant SNP in the region, and the SNP effect was in the same direction for all three traits. These traits have a high genetic correlation [3], which supports one QTL affecting these three short chain fatty acids. This SNP is located in *acyl-CoA synthetase short-chain family member 2 *(*ACSS2*), which activates acetate for de novo fatty acid synthesis [1]; thus, *ACSS2 *is a good candidate gene for a QTL with an effect on C6:0, C8:0, and C10:0. Given that this particular significant SNP is located in an intron (between exon 16 and 17) of *ACSS2*, this SNP is not likely the causal mutation, but it can be in high LD with the causal mutation. In addition, the region also had an effect on C14:1 and C16:1. The SNP effects for C14:1 and C16:1 were in the same direction, but these effects were in opposite direction of SNP effects on C6:0-C10:0. The SNP in *ACSS2 *had no significant association with C14:1 or C16:1, which indicates an additional QTL with an effect on these unsaturated fatty acids.

On BTA 27, region 27 was associated with C14:0, C16:0, C10:1, C12:1, C14:1, and C16:1 (Table 3). The region on BTA 27 includes *1-acylglycerol-3-phosphate O-acyltransferase 6 *(*AGPAT6*), which is the most abundant isoform of all *AGPAT *mRNA (~60%) in the mammary gland and involved in positioning fatty acids on the second position of the triglyceride backbone [1]. Given that 62.2% of C14:0 and 43.1% of C16:0 is located at the second position of the triglyceride backbone [39], this gene might be a candidate for this association. The SNP effects on C14:0 and C16:0 were in opposite directions, which suggests competition between C14:0 and C16:0 for the second position of the triglyceride backbone as an explanation for this association. In *AGPAT6 *knock-out mice, the composition of the triacylglycerol is altered and contains proportionally more polyunsaturated fatty acids at the expense of monounsaturated fatty acids [40], which might explain the effect of this region on the monounsaturated fatty acids. In general, the SNP effects on C10:1, C12:1, C14:1, and C16:1 were in the same direction.

### Variance explained

Table 3 shows that regions 14a and 26 explained a large portion of the total additive genetic variation of C10:1, C12:1, C14:0, C14:1, C16:0, C16:1, C18:1, and CLA. This variation is caused by *DGAT1 *and *SCD1*. For other traits, however, no regions had such large effects. The sum of the total additive genetic variance explained by the most significant SNP per region for C4:0-C12:0 and C18:0 was less than 20% of the total additive genetic variance. This finding suggests that these traits are influenced by many genes with small effects. Regions explaining roughly 1% of the total additive genetic variation or more were detected by the studied design (e.g., C10:0 on BTA1), but additional regions with either undetectable small effects or that were not dense enough in our marker set to detect the effect is likely.

Even though short chain fatty acids are produced by de novo synthesis, less than 20% of the total additive genetic variance was explained by the analyzed regions. *FASN *plays an important role in de novo synthesis, but less than 6.5% of the total additive genetic variance of short chain fatty acids is explained by the region harboring *FASN*. De novo synthesis of fatty acids requires several compounds in addition to *FASN *enzymes to elongate fatty acids. One of the compounds is acetate, which is activated by ACSS2 for de novo synthesis, *ACSS2 *was indicated as a candidate gene associated with C6:0-C10:0 in this study. More genes like this that assist FASN in de novo synthesis and, therefore, explain a portion of the total additive genetic variation is likely. Also, genes involved in transport and triacylglyceride production might explain some of the total additive genetic variation.

## Conclusions

A genome-wide association study of 50,000 SNPs was performed for milk fatty acids, resulting in many QTL. All over the genome regions were associated with milk fatty acids, some regions with just one fatty acid and other regions with multiple fatty acids. Milk fat composition is strongly influenced by polymorphisms in *DGAT1 *and *SCD1*, genes that have large effects on medium chain fatty acids and unsaturated fatty acids. Several regions showed associations with these milk fatty acids, but with smaller effects. The short chain fatty acids, C12:0 and C18:0, are not strongly affected by genes with large effects, but are influenced by regions with small effects. Some regions included candidate genes involved in milk fat synthesis pathways. On BTA 19, there were several genes involved in fat synthesis underlying the region associated with multiple fatty acids. Only in a few cases was an SNP associated with fatty acids actually located in a candidate gene. Regions identified in this study can be fine mapped to find causal mutations. The results also create opportunities for changing milk fat composition through breeding by selecting individuals based on their genetic merit for milk fat composition, which can be retrieved from the estimated SNP effects and the individual's genotype.

## Authors' contributions

ACB carried out the analysis, prepared and drafted the manuscript. HB participated in the design of the study, the coordination and helped to draft the manuscript. MHPWV helped to draft and edit the manuscript. JAMA initiated and established the overall project design. All authors read and approved the final manuscript.
